# Bilateral Intra-Articular Radiofrequency Ablation for Cervicogenic Headache

**DOI:** 10.1155/2017/1483279

**Published:** 2017-01-09

**Authors:** Charles A. Odonkor, Teresa Tang, David Taftian, Akhil Chhatre

**Affiliations:** ^1^Department of Physical Medicine & Rehabilitation, Johns Hopkins University School of Medicine, Baltimore, MD, USA; ^2^Department of Neurosurgery, Johns Hopkins University School of Medicine, Baltimore, MD, USA

## Abstract

*Introduction*. Cervicogenic headache is characterized by unilateral neck or face pain referred from various structures such as the cervical joints and intervertebral disks. A recent study of patients with cervical pain showed significant pain relief after cervical medial branch neurotomy but excluded patients with C1-2 joint pain. It remains unclear whether targeting this joint has potential for symptomatic relief. To address this issue, we present a case report of C1-2 joint ablation with positive outcomes.* Case Presentation*. A 27-year-old female presented with worsening cervicogenic headache. Her pain was 9/10 by visual analog scale (VAS) and described as cramping and aching. Pain was localized suboccipitally with radiation to her jaw and posterior neck, worse on the right. Associated symptoms included clicking of her temporomandibular joint, neck stiffness, bilateral headaches with periorbital pain, numbness, and tingling. History, physical exam, and diagnostic studies indicated localization to the C1-2 joint with 80% decrease in pain after C1-2 diagnostic blocks. She underwent bilateral intra-articular radiofrequency ablation of the C1-C2 joint. Follow-up at 2, 4, 8, and 12 weeks showed improved function and pain relief with peak results at 12 weeks.* Conclusion*. Clinicians may consider C1-C2 joint ablation as a viable long-term treatment option for cervicogenic headaches.

## 1. Introduction

Cervicogenic headaches are characterized by unilateral neck or face pain, referred from various structures such as the suboccipital muscles, cervical ligaments and joints, and intervertebral disks [1, 2]. It is estimated that about half of patients with a history of chronic neck pain after whiplash have pain originating from the cervical facet joints [2]. More recently, there has been an increased focus on the lateral atlantoaxial joint as a pain generator in patients with chronic neck pain and cervicalgia [1–3]. In asymptomatic human volunteers, distention of the lateral atlantoaxial joint with contrast generated pain at the C1-2 segmental level [3]. Many studies have shown that direct intra-articular steroid injections into the lateral atlantoaxial joint capsule can provide significant pain relief [1, 4]. With the onset of chronic joint pain, conservative measures become suboptimal for pain relief warranting a need for interventional pain procedures [5, 6]. Although invasive approaches are associated with complications, they are conducive for direct and effective individualized treatments with the proper technique [1–5].

A recent study highlighted the effectiveness of radiofrequency neurotomy of the cervical dorsal rami in relieving pain in patients with chronic C3-4 and C6-7 zygapophyseal joint pain [6]. However, patients with higher-level cervical joint pain were excluded due to the technical difficulty of the procedure [6, 7]. A more recent prospective study of 104 patients with cervical pain showed significant pain relief after cervical medial branch neurotomy [7]. However, patients with C1-2 joint pain were excluded [7]. Given suggestions of a role for targeted C1-2 joint interventions to address neck pain in the literature, we present a case report assessing the effectiveness of C1-2 intra-articular joint ablation for alleviating cervicogenic headaches. One main distinction between the intra-articular procedure versus radiofrequency neurotomy lies with the target of the lesion. In the former, the lesioning needle is inserted into the target joint and placement confirmed with a joint arthrogram. Conceptually, the intra-articular joint ablation leads to pain relief via destruction of articular cartilage or joint capsule innervated by sensory fibers, which serve as pain generators. In contrast, with radiofrequency neurotomy, the RF needle is directed alongside the target, which often tends to be medial branch nerves, or, in rare cases, the lateral branch nerves supplying the facet joints. Lesioning of the nerve occurs via coagulative necrosis. Both approaches ultimately work to disrupt pain transmission from the pain source to the brain. This report adds to the literature highlighting utility of radiofrequency ablation as a promising intervention for cervicogenic headaches.

## 2. Case Presentation

A 27-year-old female with chronic neck pain and cervicogenic headache since 2007 presented to our clinic with chief complaint of worsening neck pain. Initial onset of symptoms was noted eight years ago with status after a motor vehicle collision in which she sustained a whiplash neck injury. She reported experiencing excruciating periorbital and neck pain with unilateral right arm weakness at the time. Her symptoms deteriorated to the point where she underwent a C4–C6 laminectomy in 2009. She regained strength in her right upper extremity postoperatively. However, she continued to report neck stiffness. In 2013, she sustained a concussion after a second motor vehicle collision, further exacerbating her neck pain and headaches. She subsequently underwent several procedures including third occipital nerve blocks, cervical epidural steroid injections, and multilevel cervical facet joint injections (C2–C5), but with little to no pain relief. On presentation to our Spine Pain Center, her pain was 9/10 by visual analog scale and described as cramping, burning, and aching in quality. Pain was localized suboccipitally with radiation to her jaw and posterior aspect of her neck, worse on the right than left, and aggravated by postural changes and neck rotation.

Neck cavitation, sleep, and NSAIDs provided mild pain relief; otherwise, the pain was unrelenting. Associated symptoms included clicking of her temporomandibular joint, neck stiffness, teeth clenching, bilateral headaches with periorbital pain, numbness and tingling, anxiety, and fatigue. Pertinent negatives included no chest pain, fever, odynophagia, photophobia, syncope, dysphagia, diplopia, and blurry vision, weakness, or weight loss. She worked on a desk computer as a home sales agent. Four weeks prior to clinic visit, she noted severe functional limitations in driving, talking, eating, and work-related tasks. She was on a regimen of anxiolytics, tricyclic antidepressants, topical analgesics, and opioids but with suboptimal pain control. Conservative treatments including physical therapy, relaxation, and biofeedback failed to provide pain relief.

### 2.1. Investigations

On physical examination, she was noted to have tenderness to palpation of the proximal aspect of the right sternocleidomastoid muscle and suboccipital region and mild scapular winging (left > right) and pain with active neck rotation. Cervical flexion rotation test was positive and maximal side bending of the ipsilateral cervical spine with contralateral rotation in a chin-tuck position reproduced the pain at the AA joint. Neurological testing for cranial nerves, deep tendon, and Hoffman's reflexes was normal. Modified Sharp Purser test was negative for atlantoaxial instability. Spurling's and lateral shear testing were also normal except for bilateral paresthesias in her fingers.

**Figure 1:**
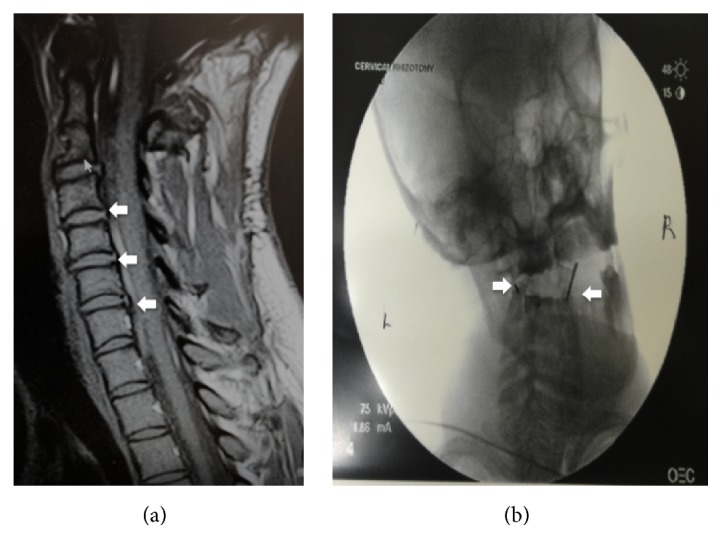
(a) MRI of the cervical spine showed degenerative changes observed at C2 (thin arrow). Multilevel disc desiccation at C2–C5 without disc height loss; C2-C3 and C3-C4 disc bulge with uncovertebral joint hypertrophy and spinal canal stenosis (fat arrows) were noted. (b) Open-mouth view of atlantoaxial joint confirmed bilateral intra-articular RFN needle placement (arrows) into the AA joint.

Her baseline Neck Disability Index Total Score was 25/50 suggesting moderate to severe disability. Magnetic Resonance Imaging of the cervical spine was positive for degenerative changes in the cervical spine, multilevel disc desiccation at C2–C5 without disc height loss, C2-C3 and C3-C4 disc bulge with uncovertebral joint hypertrophy and bilateral neural foraminal narrowing, and spinal canal stenosis (Figure 1(a)). Differential diagnosis considered at this time included cervical facetogenic pain, cervical radiculopathy, and degenerative zygapophyseal joint arthropathy. Recent diagnostic blocks up the cervical spine at C2-3 and C3–5 2 did not yield any pain relief. Combined with physical exam and clinical history, her pain was localized to the C1-2 joint.

She subsequently underwent bilateral C1-C2 intra-articular joint diagnostic “blocks” with 1% lidocaine and noted 75–80% relief in pain score. A second follow-up confirmatory block was performed at a different time, again with 75% pain relief from baseline. She was then scheduled for bilateral thermal radiofrequency ablation of the C1-C2 joint since intra-articular anesthetic blockade of the joint was both diagnostic and therapeutic. The rationale for the RFA was to obtain longer-lasting pain relief with intra-articular joint lesioning. After informed consent, the procedure was performed in aseptic fashion under fluoroscopic guidance, with patient in prone position. The beam of the C-arm was oriented to expose the atlantooccipital and axial joints. Asking the patient to open her mouth allowed an open-mouth view for clear visualization of the joints.

A 22-G RFN needle with 5 mm active tip was inserted in the neck targeting the posterior aspect of the lateral third of the inferior articular process of the atlas. This allowed for access to the joint and avoids contact with the C2 spinal nerve and ganglion. Contacting bone allowed safe depth of needle insertion to be established. Lateral and anterior views were obtained to confirm placement. The needle was then slightly withdrawn and reinserted carefully into the joint (Figure 1(b)). To confirm intra-articular placement, a joint arthrogram using a small amount of contrast medium (0.5 cc) was obtained. There was no spread into the epidural space, laterally or posteriorly. Aspiration did not reveal blood or CSF fluid. After sensory and motor testing at 0.6 volts and confirming absence of motor fasciculations or radicular symptoms with stimulation, lesioning was performed at temperature of 90°C with total lesion time of 150 s (25 s ramp time and 125 s burn time).

After lesioning, a 1 mL mixture of 0.5% bupivacaine and 8 mg/mL of triamcinolone was injected in the lesioned area with the goal to decrease postprocedure pain. Immediately after the procedure, the patient reported headaches but denied blurry vision, dysgeusia, or other associated symptoms. Her symptoms improved with fluid hydration. She was monitored for an hour after procedure and discharged without any signs of pain on lateral neck rotation. Follow-up occurred at 2, 4, 8, and 12 weeks to compare baseline and postprocedure scores in the following functional measures: Neck Disability Index (NDI), Visual Analog Scale for pain (VAS), and the RAND 36-Item Short Form Health Survey (SF-36) for health related quality of life. The minimal clinically detectable change for NDI is reported as 5 points or 10% points [8]. Since there are no established clinically meaningful changes in SF-36, an arbitrary cut-off of 50% improvement from baseline scores was used as indicator of treatment success. The patient was also referred to physical therapy and neuropsychology for exercise and behavioral pain management interventions.

### 2.2. Treatment Outcome

#### 2.2.1. Visual Analog Scores

**Figure 2:**
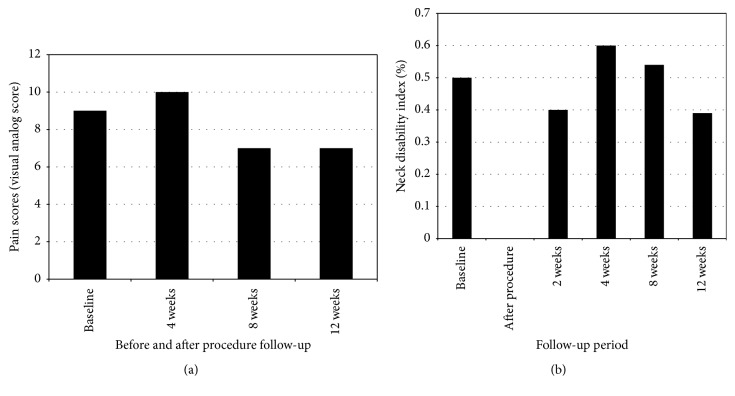
(a) Changes in visual analog pain score before procedure, and up to 12 weeks after procedure. (b) Changes in neck disability index, given as a percentage, before procedure and up to 12 weeks after procedure. A decrease of 10% from baseline score was used as an indicator of treatment success.

Using the visual analog score, the patient reported a pain score of 9 at baseline. The pain score peaked 4 weeks after the procedure. By 8 and 12 weeks after the procedure, she rated her pain score lower than at baseline and with the most optimal score observed at week 12 (Figure 2(a)) with score of 7.

#### 2.2.2. Neck Disability Index (NDI)

Baseline neck disability index was reported to be 25 at baseline. Similarly, neck disability index measures peaked at 4 weeks. However, NDI continued to decrease, with a clinically meaningful difference detected at week 12 (Figure 2(b)).

#### 2.2.3. SF-36

**Figure 3:**
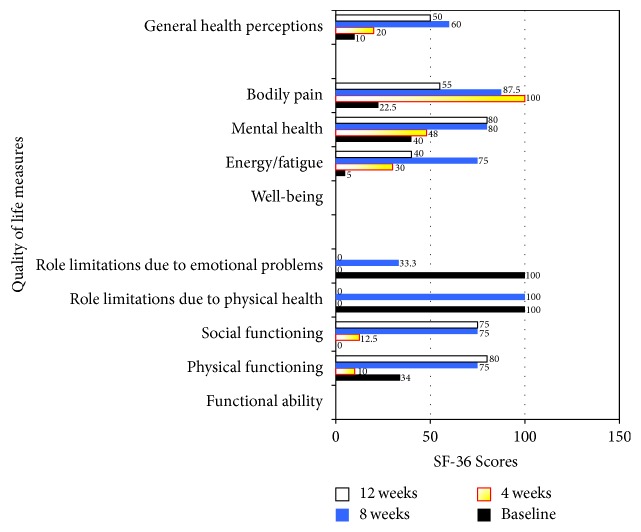
SF-36 scores in quality of life measures, before procedure and up to 12 weeks after procedure. A 50% improvement from baseline scores was used as an indicator of treatment success.

The patient noted improvement in all areas except for role limitation due to physical health and emotional problems (Figure 3).

## 3. Discussion

Of several potential etiologies of cervicogenic headache, chronic AA joint pain presents a treatment challenge [1, 2]. This is partly due to the complex anatomy of the AA joint, a tripartite joint located below the atlantooccipital joint; it is a synovial joint with an articular capsule surrounded by synovial membrane and cartilage [1–3]. It consists of three synovial joints and is the most mobile spinal articulation in the body [1, 2]. The median part of the AA joint, the pivot, reflects articulation of the dens of the axis with the atlas and the transverse ligaments. The lateral AA joint is concave in the anterior-posterior direction and allows head and neck rotation [1, 2]. Diagnosis of AA joint pain is primarily clinical and imaging studies of this joint are usually negative but are performed to rule out other more severe etiologies of neck pain such as malignancy.

It must be noted that the AA joint is a delicate area for targeted interventions and absolute care must be taken to avoid injury to proximate vascular structures. Compared to cooled RFA (performed at 60°C), conventional RFA produces elliptical rather than spherical lesions [1, 9]. In cooled RFA, water running through the probe cools the tip, which results in larger lesions. The cooling water allows lower temperatures than can be achieved by conventional RFA. In both approaches, determinants of lesion size include probe size, electrode temperature, power and voltage selection, duration of exposure, and tissue morphology [9, 10]. Stepwise regulation of the radiofrequency power and exposure produces well-demarcated lesions with controlled lesion growth.

The electrodes placed on the patient's body establish a circuit with a small electrical field at the electrode tip with alternating current causing movement of ions in tissues surrounding and adjacent to the catheter tip. This produces heat in the tissue and induces coagulative necrosis. Internally cooled probes serve as a heat sink by dissipating heat away from tissues immediately next to the probe. For bipolar radiofrequency heat lesions, the tip diameter, length, temperature, time, orientation, positioning, and tip spacing may influence the size. Further details of factors influencing lesion size are summarized elsewhere, but it is important for pain practitioners to note certain best practices to reduce risk of associated complications [9–11].

Radiological confirmation of electrode placement is key [1, 11]. Placement of electrodes parallel rather than perpendicular to the target has been shown to achieve more optimal coagulation. Sensory and motor guided stimulation help to safely identify symptomatic (pain transmitting) versus asymptomatic (non-pain transmitting) targets. The proper combination of RF-system and electrode length allows one to individually adapt the shape and volume of the generated coagulation necrosis to the target lesion [9–11]. The technical difficulty of the intra-articular AA radiofrequency ablation may be tempered by the fact that it offers a promising treatment option in experienced hands. All structures innervated by the C1, C2, and C3 segmental nerves can serve as pain generators in cervicogenic headache [1, 8, 9].

In the absence of a consensus approach to treatment, the prudent practitioner may resort to symptomatic intervention. In a reported case series by Bovaira et al., bilaterally targeting the DRG of C2 and C3 provided satisfactory relief of cervicogenic headache [12]. Others have applied pulsed RF to the greater occipital nerve and left atlantoaxial joint region with good results [9, 11]. Nonetheless, there is more evidence supporting targeting the medial branch of the posterior root of the cervical spinal nerves rather than the dorsal ganglion of roots C2 and C3 [1, 9]. The window to access the posterior capsule of the C1-2 joint may be just a few millimeters between the medial DRG and the lateral vertebral artery, using the thermal approach with a small needle and active short tip permits for a smaller lesion. We therefore caution that the approach to intra-articular RF of AA joint, whether by conventional or cooled methods, be done with cognizance of important neurovascular and anatomic structures to avoid, which will lessen the risk of complications [12, 13].

As discussed above, although patients with chronic neck pain could have multiple pain generators [2–5], the clinical history, physical exam, and diagnostic findings in this case report support AA joint as a source of pain cervicogenic headache. In addition, the patient responded with an 80% decrease in pain after C1-2 diagnostic blocks. Overall, the patient responded favorably to ablation of the C1-2 joint, as measured by the NDI, pain scores, and the SF-36 with the exception of role limitations due to physical health and emotional problems. The initial increase in NDI scores and VAS at week 4 could potentially be due to the pain from the procedure itself. A randomized controlled study by Vernon and Mior, where patients with cervicogenic headaches underwent neurotomy of C2–C6 facet joints versus sham procedure, showed that patients undergoing the procedure had an increased incidence of immediate side effects, notably, worsening tenderness and neck pain [14].

The patient continued to experience improvement in her NDI scores, with clinically meaningful improvement at 12 weeks. We suspect that intra-articular joint ablation leads to pain relief via destruction of articular cartilage innervated by sensory fibers, which serve as pain generators. It is also plausible that nonspecific lesioning of adjacent structures, synovial lining, and joint capsule obliterates proximate and related sources of pain. The patient showed improvements in all areas except in markers of psychosocial and emotional aspects of the SF-36. The lack of improvement in emotional function could be secondary to baseline patient characteristics of high catastrophizing, given her history of multiple motor vehicle accidents and living with chronic pain.

Of note, although there was an overall improvement in NDI, this assessment tool does not measure psychosocial and emotional aspects of neck disability. A prior report hints at the plausible impact of psychosocial and emotional aspects of chronic pain and the effect on a patient's overall sense of wellbeing and self-reported pain scores [15]. Importantly, there is a growing awareness of the reciprocal relationship between other factors such as insomnia and pain. It is estimated that about 60% of pain clinic patients attribute insomnia to pain, and the evidence suggests that poor sleep can decrease the pain threshold [15, 16].

Chronic pain patients with poor sleep report higher levels of anxiety and depression [15]. Insomnia is not captured by the VAS, SF-36, or NDI and could be a confounder limiting improvement in the patient's psychosocial and emotional functioning. On further discussions with our patient, she reported insomnia due to pain interference. We recommended sleeping aids, cognitive behavioral therapy, and follow-up with her primary care physician for further management. Per follow-up report adjunctive measures facilitated better coping strategies for her pain. As noted elsewhere, a multidisciplinary approach to pain including CBT and sleep management before and after procedure could help with overall outcomes in patients with chronic pain, with or without insomnia [16, 17].

A limitation of this case report is that other pain confounders such as depression and insomnia were not assessed. It also highlights some potential limitations of joint ablation in patients with chronic neck pain with high catastrophizing and other maladaptive pain behaviors. In addition, the findings from a singular case may not generalize to other patients. Nonetheless, adjunctive therapies for interventional pain procedures that address psychosocial and emotional factors in chronic pain could prove helpful for managing patients with chronic neck pain [17–19]. A proactive approach addressing the difficult but critical psychosocial aspects of chronic pain prior to procedural interventions may prove instructive to the pain management process.

Overall, ablation of the C1-C2 joint is a procedure that could result in a temporary increase in pain within a month of the procedure but results in functional improvements at 3 months. It is a procedure that, in the right hands, offers the possibility of long-term relief of cervicogenic headache that is refractory to more conservative measures. Incorporation of a multidisciplinary approach to pain before and after procedure could potentially help improve outcomes for patients with cervicogenic headaches.
